# The Folly of Anti-Vaccinators

**Published:** 1884-03

**Authors:** 


### The Folly of Anti-Vaccinators.

How any intelligent man can deliberately oppose vaccination in the face of the abundant testimony of its efficacy is truly a hard problem to solve. One of its most violent opponents in England, says the *Medical and Surgical Reporter*, was a certain William Scott, of Rotherhithe. Recently smallpox broke out in his family, and carried off his wife and three children. Regret for what might have been prevented so preyed on this man's mind that he committed suicide.

When we realize what great publicity has been given to the facts concerning vaccination, we can hardly help but lay the deaths of this man's wife and children at his door. Of course, no doubt, he was conscientious in his opposition to vaccination; but still, facts do not admit of argument, and it is almost impossible to conceive of any intelligent man finding sufficient evidence to warrant him in opposing this beneficent discovery. This sad occurrence should be given all possible publicity, as a warning to other anti-vaccination agitators.

The Effects of Tobacco.—Dr. Troitski contributes the following conclusions to the *Annales d' Hygiene:* In non-smokers of average constitutions, the mean temperature of the twenty-four hours amounts to 36.76 degrees C. (or about 98 degrees Fahr.), and the pulse-rate 72.90 degrees. In smokers, the temperature reaches 37.02 degrees C. (98.60 degrees Fahr.), and the pulse-rate 89.90 degrees. Tobacco-smoking, therefore, raises the temperature 0.26 degree C., and the pulse-rate 16 degrees. In persons of feeble constitutions, the temperature rises 0.43 degree C., and pulse-rate 11.90 degrees. Taking a mean, tobacco may be said to raise the temperature 0.29 degree C. (nearly one degree Fahr.), and to increase the cardiac pulsations by 12.70 degrees. Representing the normal temperature at 1000 in non-smokers, in moderate smokers it rises to 1008; and whereas the pulse of the former may be taken at 1000, that of the smoker is 1180. It is by increasing cardiac pulsations that tobacco has such an injurious effect on some constitutions.

NEUROTOMY IN NEURALGIA.—The evidence of many cases, collected to throw light upon the question of union of divided nerves, shows that the ends of divided nerves tend rather to grow together than keep apart. Even when so far separated as to require suturing, they are very amenable to treatment, as Mr. Clark, of Glasgow, has shown in his article on "Immediate Suture of Divided Nerves." The practical point is this—simple neurotomy has but little, if any permanent, effect in curing neuralgia; a portion of the nerve must be dissected out, or its ends sutured into neighboring fibrous tissue, and so kept apart. Were we to take a lesson from traumatic pathology, we should tear and bruise our nerve, not cut it and treat it antiseptically; but let us remember that such a thing as Tetanus does exist, and that lacerated nerve tissue tends to promote that terrible malady.

WE hear with pleasure that our *confreres* in Paris have at last started a first-rate Dental College. Some of our readers may have noticed No. 3, Rue de l'Abbaye, an old palace, which quite refreshed the sight after the uniform row of tall white houses. Instead of the monotonous "suite" of rooms on the usual French flats, with low ceilings and too much ginger bread gilding, we found an old house with large lofty rooms, in fact, a regular ancient Italian palace--with such an enviable hospital, and men like Messrs. Andrieu Brasseur, Mordaunt Stevens, Colignon Kingsley, Galliard du Bouchet, as senior professors and clinical teachers, we have no doubt of the future success of the "Institut Odontotechnique of France."

DEATH FROM PASSION.--Very few persons realize the necessity of cultivating an equable temper, and of avoiding passion. With a weak heart or atheromatous cerebral arteries, the giving way to passion may cause a giving way of the heart or a blood-vessel, and thus prove fatal. It was the result of passion that caused John Hunter's weak heart to give out, and we should ever remember his sad fate as a warning.

Teeth at Birth—Ulceration of Tongue.—In a recent number of "l'Union Medicale," M. Jules Besnier records a case of an infant born with teeth already cut. The child suffered from coryza, and had consecutive ulcerations of its tongue. Dr. Lardier notices that he published a similar case in "Gazette Obstetricale de Paris," Aug. 15th, 1875. In his case the right lower incisor had been removed three days after the infant's birth. The presence of the tooth had rendered sucking difficult at first, but when the tongue became ulcerated, the child could not suck at all. Hæmorrhage, although said to be dangerous in such cases, is really quite exceptional, unless of course the hœmophilil tic diathesis be present These prematurely developed teeth are undoubtedly rare, but we find many cases occur which are not put on record. The teeth usually require avulsion, but when they can be left to nature, it is customary for them to be shed very early.

The opposite condition, non-eruption of teeth in children and adults, occasionally occurs. We had a case under observation not long since. The patient, a phthisical woman, was quite and congenitally edentulous. Her mother had been so, and she stated that one of her children, a girl, suffered from the same defect. It is undoubted that dental abnormalities are commonly hereditary.

